# Tetracyclines and Neuromuscular Disorders

**DOI:** 10.2174/157015912800604498

**Published:** 2012-06

**Authors:** Daniele Orsucci, Michelangelo Mancuso, Massimiliano Filosto, Gabriele Siciliano

**Affiliations:** 1 Department of Neuroscience, Neurological Clinic, University of Pisa, Italy, Via Roma; 2 Neurological Clinic, University of Brescia, Italy

**Keywords:** Doxycycline, mitochondria, minocycline, neurodegeneration, PARP-1, progressive external ophthalmoplegia, ROS, tetracycline.

## Abstract

Tetracyclines are a class of antibiotics which could act as neuroprotective molecules in several neurological disorders, such as Huntington disease, Parkinson disease, stroke and multiple sclerosis. The main biological effects of tetracyclines are the inhibition of microglial activation, the attenuation of apoptosis and the suppression of reactive oxygen species production. The anti-apoptotic effect of tetracyclines involves the mitochondrion, and the major target for neuroprotective effects of tetracyclines lies within the complex network that links mitochondria, oxidative stress and apoptosis.

Neuromuscular disorders are due to dysfunction of motor neurons, peripheral nerves, neuromuscular junction, or skeletal muscle itself. Animal studies have shown that minocycline could play neuroprotective effects in amyotrophic lateral sclerosis, but these positive findings have not been replicated in patients. Other neuromuscular disorders which tetracyclines may benefit are Guillain-Barré syndrome and other neuropathies, muscular dystrophies and mitochondrial disorders. However, well-designed double-blind controlled trials are still needed. Further studies are strongly needed to establish the most appropriate timing and dosage, as well as the indications for which tetracyclines could be effective and safe.

Here, we review the neuroprotective effects of tetracyclines in animal models, the clinical studies in humans, and we focus on their potential application in patients with neuromuscular disorders.

## INTRODUCTION

Tetracyclines are a group of broad-spectrum antibiotics including tetracycline, doxycycline, minocycline, and others. In humans, long-term treatment with tetracyclines is generally safe and well tolerated. The capacity of tetracyclines to alleviate disease for several neurological disorders, such as multiple sclerosis, neurodegenerative and neuromuscular disorders, stroke and traumatic injury, is increasingly being recognised in animal models [1, 2]. Tetracyclines may play some neuroprotective role also in human patients with central neurological diseases, including stroke [3], multiple sclerosis [4, 5], Parkinson disease [6], Huntington disease [7] and fragile X syndrome [8], but well-designed double-blind controlled trials are still lacking. The main biological effects of tetracyclines are inhibition of microglial activation, attenuation of apoptosis, and suppression of reactive oxygen species production, as deeply discussed elsewhere [1]. 

The main mode of action of tetracyclines is still unclear, but the anti-apoptotic effect involves the mitochondrion [1]. Studies reported that minocycline reduced mitochondrial Ca^2+^ uptake, stabilized mitochondrial membranes, inhibited mitochondrial release into the cytosol of cytochrome *c* (cyt *c*) and other apoptotic factors, such as AIF (apoptosis inducing factor). Other effects include up-regulation of mitochondrial bcl-2 (an antiapoptotic protein), direct scavenging of peroxynitrite [9], inhibition of mitogen activated protein kinases, and poly(ADP-ribose) polymerase-1 (PARP-1) inhibition [10]. Furthermore, tetracyclines act at the mitochondrial level to rescue the collapse of transmembrane potential and the alterations of permeability transition, both critical steps for the release of apoptogenic factors such as cyt *c*, AIF and Smac/Diablo [11]. The major target for the neuroprotective effects of tetracyclines lies within the complex network that links mitochondria, oxidative stress and apoptosis, and could involve PARP-1 direct inhibition [1]. 

Minocycline penetrates the cerebrospinal fluid better than doxycycline and other tetracyclines; therefore, neurological research mainly focused on minocycline [1]. In this article, we review the neuroprotective effects of tetracyclines in animal models, the clinical studies in humans, and we focus on their potential application in patients with neuromuscular disorders. Neuromuscular disorders are diseases due to dysfunction of motor neurons, peripheral nerves, neuro- muscular junction, or skeletal muscle itself. 

A possible role of tetracyclines on muscular atrophy has also been proposed. Shefer and co-workers [12] have reported that tetracycline, as well as exercise running, enhanced muscle recovery after atrophy. These authors examined how rat muscular satellite cells were affected by atrophic conditions induced by limb immobilization and by pharmacological (tetracycline) and physiological (exercise running) countermeasures. Differently from exercise running, tetracycline did not induce muscle mass re-gain [12]. However, tetracycline had a beneficial effect on muscle cells, as it enhanced the expression levels of muscle specific regulatory factors, essential for proliferation and differentiation (myogenesis), even after prolonged periods of muscle disuse [12]. The association between tetracycline and exercise may be interesting, and should be tested in animal models of muscle atrophy and disease.

## AMYOTROPHIC LATERAL SCLEROSIS

Amyotrophic lateral sclerosis (ALS) is a motor neuron disease, with selective degeneration of the anterior horn cells of the spinal cord and cortical motor neurons. Approximately 90-95% of cases are sporadic, and <10% familial. About 20% of familial cases result from mutation in the gene encoding for the Cu/Zn-superoxide dismutase (*SOD1*). The aetiology and pathogenesis of the sporadic form of the disease are poorly understood; mitochondrial dysfunction and oxidative stress are involved [13, 14]. Mice expressing mutant *SOD1 *provide an animal model of ALS. Early studies had shown that minocycline could delay disease onset and extend survival in ALS mice [11], and prevent neurotoxicity induced by cerebrospinal fluid from ALS patients [15]. 

More recently, a mouse model for live imaging of neuroinflammatory responses in ALS (GFAP-luc/SOD1 [G93A] reporter mouse) allowed to analyze in real time the effects of minocycline treatment initiated at different stages of the disease [16]. Unlike neuroprotection that was conferred when minocycline was administered pre-symptomatically, treatment with minocycline initiated after the disease onset significantly altered glial responses and exaggerated neuroinflammation [16]. This suggests that when administered at later stages of disease, once microglial cells are chronically reactive, minocycline may not have anti-inflammatory properties, and may alter astrocyte reactivity and increase microgliosis [16]. Moreover, it has been shown that the combination of minocycline (170 mg/kg) and riluzole (10 mg/kg) could induce some motor impairment in mice (the time spent on the rod was significantly decreased in the group treated for 5 days with the riluzole/minocycline combination compared to the group that received riluzole alone) [17]. Riluzole is the only available therapy for ALS, with only marginal effects on disease survival; it may inhibit the release of glutamate and may block some of the postsynaptic effects of glutamate [17].

A pilot study showed that combined treatment with minocycline and riluzole was safe in human patients with ALS [18]. A multicenter, randomized placebo-controlled phase III trial could not replicate in patients the positive laboratory findings [19]. After a 4-month lead-in phase, 412 patients were randomly assigned to receive placebo or minocycline in escalating doses of up to 400 mg/day for 9 months [19]. Clinical deterioration was faster in the minocycline group than in the placebo group [19]. Post-hoc analyses suggested that the outcome was not related to dose, caused by adverse events, or due to interactions with riluzole [19]. Therefore, the safety of minocycline in ALS patients is still an open question, and the possibility that minocycline may be harmful must be kept in mind when considering its use in neurological practice [1, 2].

## NEUROPATHIES

Experimental autoimmune neuritis (EAN) is a widely used animal model of the human acute inflammatory demyelinating polyradiculoneuropathy, which is the most common subtype of Guillain-Barré syndrome (GBS). Zhang and co-workers [20] found that minocycline, immediately post immunization, significantly attenuated the severity and duration of rat EAN. Macrophage and T-cell infiltration and demyelination in sciatic nerve were also significantly reduced, as well as mRNA expression of metalloproteinase-9, inducible nitric oxide synthase and proinflammatory cytokines. Furthermore, minocycline attenuated mechanical allodynia and suppressed microglial activation in spinal cord [20]. Very recently, the same group obtained similar results with doxycycline 40 mg/kg from the day 9 to day 14 post immunization [21].

Furthermore, administration of minocycline (40 and 80 mg/kg, i.p.) for 2 weeks started 2 weeks after diabetes induction attenuated the development of diabetic neuropathy in streptozotocin-induced diabetic rats [22]. These beneficial effects of minocycline were partly mediated by its anti-inflammatory effect by reducing the levels of proinflammatory cytokines and in part by modulating oxidative and nitrosative stress [22]. 

Interestingly, minocycline has been reported to have some beneficial effects in animal models of nerve-injury induced neuropathic pain [23], mechanical allodynia and central sensitization following peripheral second-degree burn injury [24], oxaliplatin-induced neuropathic pain [25], paclitaxel-induced allodynia [26], formalin-induced nociception [27]. It has also been reported that minocycline could exert analgesia peripherally through sodium channel inhibition in the primary afferent neurons, as well as centrally through microglial inhibition in the spinal cord [28], and that it could prevent impaired glial glutamate uptake in the spinal sensory synapses [29] in animal models. Moreover, the injury-induced activation of microglia and leukocytes and the subsequent activation of neuropeptides involved in nociception processes (prodynorphin and pronociceptin) may be potential targets for the attenuation of neuropathic pain by minocycline, as observed in a rat model of neuropathic pain [30]. Despite these apparently pleiotropic effects of tetracyclines in animal models of GBS and other neuropathies, no clinical trials are available to date.

## MUSCULAR DYSTROPHIES

Muscular dystrophies are a heterogeneous group of disorders for which there are currently no effective treatments. In a transgenic mouse model of oculopharyngeal muscular dystrophy, manifesting progressive muscle weakness accompanied by intranuclear aggregates, the onset and severity of these abnormalities were substantially delayed and attenuated by doxycycline treatment [31]. 

More recently, Girgenrath and co-workers [32] reported that doxycycline was effective in a laminin-α2 (*Lama2*)-null model of congenital muscular dystrophy. Human congenital muscular dystrophy type 1A is an autosomal recessive disease caused by loss-of-function mutations in *Lama2*, and results in motor nerve and skeletal muscle dysfunction. The time at which half of *Lama2*-null mice died increased from approximately 32 days for the untreated cohort up to approximately 70 days for the doxycycline-treated mice [32]. Minocycline similarly increased the lifespan [32]. Oral doxycycline improved postnatal growth rate and delayed the onset of hind-limb paralysis [32]. Doxycycline-treated mice also had more and larger myofibers [32]. Furthermore, doxycycline decreased muscular inflammation, increased Akt phosphorylation, and decreased apoptosis markers, such as Bax and activated caspase-3 [32]. Very recently, the same group [33] also determined how nerve pathology was affected by doxycycline treatment, observing that myelinating Schwann cells were significantly increased in doxycycline-treated compared with untreated sciatic nerves [33]. In addition, doxycycline-treated peripheral nerves had significantly less pathology as measured by assays such as amount of unmyelinated or disorganized axons [33].

No clinical trials of tetracyclines in muscular dystrophies are available to date.

## MITOCHONDRIAL DISORDERS

Tetracyclines could be also protective in mitochondrial diseases. Mitochondrial disorders are caused by impairment of the mitochondrial respiratory chain [34]. They are one of the most common inherited neuromuscular disorders, with an estimated prevalence of >1/10,000 [35]. The effects of mutations affecting the respiratory chain may be multisystemic, with involvement of visual and auditory pathways, heart, central nervous system, and skeletal muscle [34]. Oxidative stress biomarkers may be useful to detect redox imbalance in mitochondrial diseases and to provide non-invasive tools to monitor disease status [36]. Despite great progress in the molecular understanding of mitochondrial diseases, the treatment of these disorders is still inadequate [37].

A study of cybrid cells bearing the mitochondrial DNA (mtDNA) 11778 mutation, associated with Leber’s hereditary optic neuropathy, showed that minocycline increased the survival of these cells after calcium overload [38]. In the cytoplasmic hybrid (“cybrid”) technique, culturable cells depleted of endogenous mitochondrial DNA are repopulated with mitochondria (with their own mitochondrial DNA) from patients. In “Leber” cybrids, the mitochondrial membrane potential was significantly conserved and the active-caspase-3/procaspase-3 ratio was decreased by minocycline [38]. 

Moreover, it has been reported an improvement in ocular motility in a patient with ocular mitochondrial myopathy (progressive external ophthalmoplegia) following treatment with tetracycline [39]. For this reason, our group performed a double-blind randomized pilot study (followed by an adjunctive open-label phase) in order to evaluate if tetracycline (500 mg/day x 14 days/month x 3 months) could be useful in patients (n = 16) with progressive external ophthalmoplegia [40]. Our results did not formally support any effect of tetracycline on eye motility, but some possible protective effects could not be completely ruled out. i.e., a further analysis suggested a possible difference between the tetracycline group and the placebo group, significant at least for oblique motility, when comparing the ratio between the end of the double-blind phase and baseline [40]. Furthermore, we observed that tetracycline could modify some oxidative stress biomarkers in these patients (namely total glutathione and advanced oxidation protein products). Moreover, a significant reduction of lactate levels during the open-label phase has been noted, which could suggest that an improvement in aerobic metabolism could accompany the reduced oxidative stress levels [40]. These data support a possible action of tetracycline (direct or indirect) on oxidative stress and mitochondrial metabolism, at least in patients with progressive external ophthalmoplegia. To our knowledge, this has been the first report of antioxidant action of a tetracycline in humans. Further studies are needed in order to confirm these effects of tetracycline, and to clarify the mechanisms of action for protective effects of tetracyclines in mitochondrial disorders, if any. 

## CONCLUSION

Despite the numerous studies reporting possible neuroprotective effects of tetracyclines, there is ongoing debate on the presence of unproductive effects of this class of antibiotics (Table **1**). Minocycline may have variable and even deleterious effects in different species and models according to the mode of administration, dose and timing, and it is important to examine this drug carefully before clinical use in humans [1]. 

As already reported, a randomized trial could not replicate in ALS patients the positive findings from laboratory studies [19]. Clinical deterioration was faster in the minocycline group than in the placebo group [19]. In animal models, treatment with minocycline initiated after the ALS onset had detrimental effects, suggesting that the neuro-protective effects may be limited to the presymptomatic stages of the disease [16]; furthermore, the combination of minocycline and riluzole could induce some motor impairment [17]. Therefore, at this stage, minocycline treatment is not warranted in patients with ALS.

Some concerns may also be justified in mitochondrial disorders. It has been reported the case of a teenager in which mitochondrial myopathy with severe lactic acidosis had presented following mononucleosis and minocycline use [41]. In our trial, a patient developed fully reversible creatine kinase elevation [40]. Moreover, it has been observed that minocycline at low concentrations could impair several energy-dependent functions of mitochondria *in vitro* and trigger mitochondrial swelling and cyt *c* release [42]. Minocycline-dependent swelling was associated with mitochondrial depolarization [42]. Furthermore, mitochondrial translation in cells with normal mitochondrial function was inhibited by doxycycline and tetracycline (which target the prokaryotic ribosome for inhibition), and cells from patients with mitochondrial translational defects were more sensitive to doxycycline and tetracycline-induced mitochondrial translation inhibition [43]. Further studies are needed to establish the real safety of tetracyclines in patients with mitochondrial disorders. 

The potential clinical utility of the neuroprotective effects of tetracyclines is still a matter of debate, with contradictory evidence ranging from neuroprotection to the exacerbation of toxicity in various experimental models and human trials [44], and further studies are strongly needed to establish the most appropriate timing and dosage, as well as the indications for which tetracyclines could be effective and safe. The development of new chemically modified tetracyclines without antibacterial activity [45] is of great interest also, and may represent a prerequisite for large scale human utilization. 

Because of the epidemiological relevance of neuro-muscular disorders, further studies are needed to clarify the role of tetracyclines in such conditions. Furthermore, these studies may help elucidate the mechanism behind the mitochondrial dysfunction detectable in neurodegeneration, and may be of relevance for the development of strategies in the treatment of these disorders.

## Figures and Tables

**Table 1.** Selected Preclinical and Clinical Studies with Tetracyclines in Neuromuscular Disorders

Disease	Animal Model Studies	Human Studies	Conclusions
ALS	Minocycline could delay disease onset and extend survival [11]. No neuroprotection and possible detrimental effects when the treatment was initiated after the disease onset [16].	A multicenter, randomized placebo-controlled phase III trial on 412 patients showed faster deterioration in the minocycline group [19].	At this stage, minocycline treatment is not warranted in patients with ALS. Further preclinical studies still needed.
GBS	Minocycline and doxycycline significantly attenuated the severity and duration of rat experimental autoimmune neuritis [20-21].	Not available.	Clinical trials and further preclinical studies still needed.
Muscular dystrophies	Beneficial effects in animal models of oculopharyngeal muscular dystrophy [31] by doxycycline, and in congenital muscular dystrophy type 1A by doxycycline and minocycline [32-33].	Not available.	Clinical trials and further preclinical studies still needed.
Mitochondrial disorders	Not available.	A double-blind randomized pilot study (followed by an adjunctive open-label phase) on 16 PEO patients did not formally support any effect of tetracycline on eye motility. Possible effects on oblique motility. Tetracycline could modify some oxidative stress biomarkers and reduce lactate levels [40].	Further preclinical and clinical studies strongly needed.

ALS, amyotrophic lateral sclerosis; GBS, Guillain-Barré syndrome; PEO, progressive external ophthalmoplegia.

## ABBREVIATIONS

AIF: = Apoptosis inducing factor
ALS: = Amyotrophic lateral sclerosis
EAN: = Experimental autoimmune neuritis
GBS: = Guillain-Barré syndrome
PARP-1: = Poly(ADP-ribose) polymerase-1
SOD1: = Cu/Zn-superoxide dismutase

## References

[1] Orsucci D, Calsolaro V, Mancuso M, Siciliano G (2009). Neuroprotective effects of tetracyclines: molecular targets, animal models and human disease. CNS Neurol. Disord. Drug Targets.

[2] Yong VW, Wells J, Giuliani F, Casha S, Power C, Metz LM (2004). The promise of minocycline in neurology. Lancet Neurol.

[3] Lampl Y, Boaz M, Gilad R, Lorberboym M, Dabby R, Rapoport A, Anca-Hershkowitz M, Sadeh M (2007). Minocycline treatment in acute stroke: an open-label, evaluator-blinded study. Neurology.

[4] Metz LM, Zhang Y, Yeung M, Patry DG, Bell RB, Stoian CA, Yong VW, Patten SB, Duquette P, Antel JP, Mitchell JR (2004). Minocycline reduces gadolinium-enhancing magnetic resonance imaging lesions in multiple sclerosis. Ann. Neurol.

[5] Zabad RK, Metz LM, Todoruk TR, Zhang Y, Mitchell JR, Yeung M, Patry DG, Bell RB, Yong VW (2007). The clinical response to minocycline in multiple sclerosis is accompanied by beneficial immune changes: a pilot study. Mult. Scler.

[6] (2006). A randomized double-blind futility clinical trial of creatine and minocycline in early Parkinson disease. Neurology.

[7] Bonelli RM, Hodl AK, Hofmann P, Kapfhammer HP (2004). Neuroprotection in Huntington's disease: a 2-year study on minocycline. Int. Clin. Psychopharmacol.

[8] Paribello C, Tao L, Folino A, Berry-Kravis E, Tranfaglia M, Ethell IM, Ethell DW (2010). Open-label add-on treatment trial of minocycline in fragile X syndrome. BMC Neurol.

[9] Schildknecht S, Pape R, Muller N, Robotta M, Marquardt A, Burkle A, Drescher M, Leist M (2011). Neuroprotection by minocycline caused by direct and specific scavenging of peroxynitrite. J. Biol. Chem.

[10] Tao R, Kim SH, Honbo N, Karliner JS, Alano CC (2010). Minocycline protects cardiac myocytes against simulated ischemia-reperfusion injury by inhibiting poly(ADP-ribose) polymerase-1. J. Cardiovasc. Pharmacol.

[11] Zhu S, Stavrovskaya IG, Drozda M, Kim BY, Ona V, Li M, Sarang S, Liu AS, Hartley DM, Wu DC, Gullans S, Ferrante RJ, Przedborski S, Kristal BS, Friedlander RM (2002). Minocycline inhibits cytochrome c release and delays progression of amyotrophic lateral sclerosis in mice. Nature.

[12] Shefer G, Carmeli E, Rauner G, Yablonka-Reuveni Z, Benayahu D (2008). Exercise running and tetracycline as means to enhance skeletal muscle stem cell performance after external fixation. J. Cell Physiol.

[13] Crugnola V, Lamperti C, Lucchini V, Ronchi D, Peverelli L, Prelle A, Sciacco M, Bordoni A, Fassone E, Fortunato F, Corti S, Silani V, Bresolin N, Di Mauro S, Comi GP, Moggio M (2010). Mitochondrial respiratory chain dysfunction in muscle from patients with amyotrophic lateral sclerosis. Arch. Neurol.

[14] Mancuso M, Filosto M, Orsucci D, Siciliano G (2008). Mitochondrial DNA sequence variation and neurodegeneration. Hum. Genomics.

[15] Tikka TM, Vartiainen NE, Goldsteins G, Oja SS, Andersen PM, Marklund SL, Koistinaho J (2002). Minocycline prevents neurotoxicity induced by cerebrospinal fluid from patients with motor neurone disease. Brain.

[16] Keller AF, Gravel M, Kriz J (2011). Treatment with minocycline after disease onset alters astrocyte reactivity and increases microgliosis in SOD1 mutant mice. Exp. Neurol.

[17] Milane A, Tortolano L, Fernandez C, Bensimon G, Meininger V, Farinotti R (2009). Brain and plasma riluzole pharmacokinetics: effect of minocycline combination. J. Pharm. Pharm. Sci.

[18] Pontieri FE, Ricci A, Pellicano C, Benincasa D, Buttarelli FR (2005). Minocycline in amyotrophic lateral sclerosis: a pilot study. Neurol. Sci.

[19] Gordon PH, Moore DH, Miller RG, Florence JM, Verheijde JL, Doorish C, Hilton JF, Spitalny GM, MacArthur RB, Mitsumoto H, Neville HE, Boylan K, Mozaffar T, Belsh JM, Ravits J, Bedlack RS, Graves MC, McCluskey LF, Barohn RJ, Tandan R (2007). Efficacy of minocycline in patients with amyotrophic lateral sclerosis: a phase III randomised trial. Lancet Neurol.

[20] Zhang ZY, Zhang Z, Fauser U, Schluesener HJ (2009). Improved outcome of EAN an animal model of GBS through amelioration of peripheral and central inflammation by minocycline. J. Cell Mol. Med.

[21] Yi C, Zhang Z, Wang W, Zug C, Schluesener HJ (2011). Doxycycline attenuates peripheral inflammation in rat experimental autoimmune neuritis. Neurochem. Res.

[22] Pabreja K, Dua K, Sharma S, Padi SS, Kulkarni SK (2011). Minocycline attenuates the development of diabetic neuropathic pain: possible anti-inflammatory and anti-oxidant mechanisms. Eur. J. Pharmacol.

[23] Mei XP, Xu H, Xie C, Ren J, Zhou Y, Zhang H, Xu LX (2011). Post-injury administration of minocycline: an effective treatment for nerve-injury induced neuropathic pain. Neurosci. Res.

[24] Chang YW, Waxman SG (2010). Minocycline attenuates mechanical allodynia and central sensitization following peripheral second-degree burn injury. J. Pain.

[25] Boyette-Davis J, Dougherty PM (2011). Protection against oxaliplatin-induced mechanical hyperalgesia and intraepidermal nerve fiber loss by minocycline. Exp. Neurol.

[26] Liu CC, Lu N, Cui Y, Yang T, Zhao ZQ, Xin WJ, Liu XG (2010). Prevention of paclitaxel-induced allodynia by minocycline: Effect on loss of peripheral nerve fibers and infiltration of macrophages in rats. Mol. Pain.

[27] Li K, Fu KY, Light AR, Mao J (2010). Systemic minocycline differentially influences changes in spinal microglial markers following formalin-induced nociception. J. Neuroimmunol.

[28] Kim TH, Kim HI, Kim J, Park M, Song JH (2011). Effects of minocycline on Na(+) currents in rat dorsal root ganglion neurons. Brain Res.

[29] Nie H, Zhang H, Weng HR (2010). Minocycline prevents impaired glial glutamate uptake in the spinal sensory synapses of neuropathic rats. Neuroscience.

[30] Mika J, Rojewska E, Makuch W, Przewlocka B (2010). Minocycline reduces the injury-induced expression of prodynorphin and pronociceptin in the dorsal root ganglion in a rat model of neuropathic pain. Neuroscience.

[31] Davies JE, Wang L, Garcia-Oroz L, Cook LJ, Vacher C, O'Donovan DG, Rubinsztein DC (2005). Doxycycline attenuates and delays toxicity of the oculopharyngeal muscular dystrophy mutation in transgenic mice. Nat. Med.

[32] Girgenrath M, Beermann ML, Vishnudas VK, Homma S, Miller JB (2009). Pathology is alleviated by doxycycline in a laminin-alpha2-null model of congenital muscular dystrophy. Ann. Neurol.

[33] Homma S, Beermann ML, Miller JB (2011). Peripheral nerve pathology, including aberrant Schwann cell differentiation is ameliorated by doxycycline in a laminin-alpha2-deficient mouse model of congenital muscular dystrophy. Hum. Mol. Genet.

[34] DiMauro S, Schon EA (2003). Mitochondrial respiratory-chain diseases. N. Engl. J. Med.

[35] Schaefer AM, McFarland R, Blakely EL, He L, Whittaker RG, Taylor RW, Chinnery PF, Turnbull DM (2008). Prevalence of mitochondrial DNA disease in adults. Ann. Neurol.

[36] Mancuso M, Orsucci D, Logerfo A, Rocchi A, Petrozzi L, Nesti C, Galetta F, Santoro G, Murri L, Siciliano G (2010). Oxidative stress biomarkers in mitochondrial myopathies, basally and after cysteine donor supplementation. J. Neurol.

[37] Chinnery P, Majamaa K, Turnbull D, Thorburn D (2006). Treatment for mitochondrial disorders. Cochrane Database Syst. Rev.

[38] Haroon MF, Fatima A, Scholer S, Gieseler A, Horn TF, Kirches E, Wolf G, Kreutzmann P (2007). Minocycline a possible neuroprotective agent in Leber's hereditary optic neuropathy (LHON): studies of cybrid cells bearing 11,778 mutation. Neurobiol. Dis.

[39] Omar A, Johnson LN (2007). Tetracycline delays ocular motility decline in chronic progressive external ophthalmoplegia. Neurology.

[40] Mancuso M, Orsucci D, Calsolaro V, Logerfo A, Allegrini L, Petrozzi L, Simoncini C, Rocchi A, Trivella F, Murri L, Siciliano G (2011). Tetracycline treatment in patients with progressive external ophthalmoplegia. Acta Neurol. Scand.

[41] Zoraster RM, Rison RA (2008). Severe lactic acidosis secondary to minocycline in a teenager with infectious mononucleosis and mitochondrial myopathy. Clin. Neurol. Neurosurg.

[42] Kupsch K, Hertel S, Kreutzmann P, Wolf G, Wallesch CW, Siemen D, Schonfeld P (2009). Impairment of mitochondrial function by minocycline. FEBS J.

[43] Jones CN, Miller C, Tenenbaum A, Spremulli LL, Saada A (2009). Antibiotic effects on mitochondrial translation and in patients with mitochondrial translational defects. Mitochondrion.

[44] Plane JM, Shen Y, Pleasure DE, Deng W (2010). Prospects for minocycline neuroprotection. Arch. Neurol.

[45] Gu Y, Lee HM, Simon SR, Golub LM (2011). Chemically modified tetracycline-3 (CMT-3): A novel inhibitor of the serine proteinase elastase. Pharmacol. Res.

